# Scalable Extraction of Big Macromolecular Data in Azure Data Lake Environment

**DOI:** 10.3390/molecules24010179

**Published:** 2019-01-05

**Authors:** Dariusz Mrozek, Tomasz Dąbek, Bożena Małysiak-Mrozek

**Affiliations:** Institute of Informatics, Silesian University of Technology, Akademicka 16, 44-100 Gliwice, Poland; dabek.t@gmail.com (T.D.); bozena.malysiak@polsl.pl (B.M.-M.)

**Keywords:** data processing, Cloud computing, Big Data, data extraction, data lake, parallel computing, querying, proteins, nucleic acids, macromolecules, 3D structure, structural bioinformatics

## Abstract

Calculation of structural features of proteins, nucleic acids, and nucleic acid-protein complexes on the basis of their geometries and studying various interactions within these macromolecules, for which high-resolution structures are stored in Protein Data Bank (PDB), require parsing and extraction of suitable data stored in text files. To perform these operations on large scale in the face of the growing amount of macromolecular data in public repositories, we propose to perform them in the distributed environment of Azure Data Lake and scale the calculations on the Cloud. In this paper, we present dedicated data extractors for PDB files that can be used in various types of calculations performed over protein and nucleic acids structures in the Azure Data Lake. Results of our tests show that the Cloud storage space occupied by the macromolecular data can be successfully reduced by using compression of PDB files without significant loss of data processing efficiency. Moreover, our experiments show that the performed calculations can be significantly accelerated when using large sequential files for storing macromolecular data and by parallelizing the calculations and data extractions that precede them. Finally, the paper shows how all the calculations can be performed in a declarative way in U-SQL scripts for Data Lake Analytics.

## 1. Introduction

Parsing macromolecular data files is one of the first processes preceding many operations performed on 3D structures of proteins and nucleic acids, including statistical calculations over geometry of proteins, investigations of protein inter-residue contacts, docking prediction, 3D protein structure alignment, studying intra-protein interactions that stabilize protein molecules, like disulphide bonds, aromatic-aromatic interactions, sulphur–aromatic interactions, ionic interactions. Macromolecular data describing 3D structures of proteins and nucleic acids are usually stored in files that have various formats. Protein Data Bank (PDB) [1], the most popular repository established to collect data describing 3D structures of macromolecules, stores and exchanges the data in three formats—PDB [2], mmCIF [3], and PDBML [4]. All of them are text files with sections and records that hold a particular type of information. The descriptions of macromolecular structures in these files include primarily their geometries, but also many other features that determine the physical and chemical properties of DNA, RNA, and protein molecules. These features can be directly extracted from the files or derived by calculations on the basis of macromolecular data stored in them.

However, the exponential growth of macromolecular data in the Protein Data Bank may pose pressure on the existing hardware equipment, as compute capabilities of desktop computers are usually limited [5]. With this hardware, some calculations performed over protein geometries and atomic interactions can be successfully completed in several minutes only for smaller collections of macromolecular data. However, as the repositories grow quickly every year, desktop computers may become a bottleneck in the entire discovery process [6,7]. This results in focusing on the use of various scalable platforms that would support efficient data processing and calculations [8,9,10]. Taking into account the complex nature of biological data, including macromolecular data of proteins, various storage formats for the data, the growing amount of the data, and finally, the complexity of some calculation processes performed over the data, we may find out that we meet challenges of Big Data processing and analysis [11,12,13]. Performing many calculations with 3D structures of proteins and nucleic acids meets the 5V model of Big Data at least in terms of the *volume* and the *variety* of data, and maybe for some calculations (e.g., those related to drug design) also the *value*. This leads to the use of various Big Data platforms, like Hadoop [14] or Spark [15], to perform these calculations for large collections of data, and make us switching to Cloud computing platforms that enable almost unlimited compute resources for scaling the calculations broadly [16].

In this paper, we show how parsing and extracting big macromolecular data of proteins and nucleic acids can be effectively performed in highly scalable Azure Data Lake cloud environment. The  solution presented here mitigates the problem of limited compute resources of desktop computers, in terms of data storing and processing. Moreover, by using the declarative U-SQL query language we now simplify the manipulation of macromolecular data and performing calculations over 3D structures of macromolecules. Together with the methods, in the paper, we show sample U-SQL scripts that can be used in scalable, parallel calculations performed in the Azure Data Lake environment.

## 2. Related Works

The spectrum of calculations performed over 3D structures of macromolecules, including proteins, can be very broad. It may include various exploration operations that are based on simple calculations on particular atoms, but also more complex calculations related to structure similarity searching, structural alignment, structural superposition, or computational protein structure determination through prediction. Among the tools that were reported for the exploration of various features of proteins and their geometries, there have been developed several ones that are focused on the recognition and the analysis of different types of interactions in proteins carried out on experimentally determined, high-resolution protein structures from the Protein Data Bank. For example, the protein interactions calculator (PIC) server reported by Tina et al. [17] was developed for studying different types of interactions that occur within a given protein structure. The aromatic-aromatic Interactions Database (A2ID) reported by Chourasia et al. [18] allows studying the aromatic-aromatic networks within proteins. IntGeom [19] can be used for the calculation of interaction geometry between planar groups in proteins. Mentioned examples, however, neither focus on the performance of the operations nor provide large flexibility on performed data explorations.

In this regard and in terms of big data processing, interesting ideas were proposed by Hazelhurst in [20]. In the work, the author reported a Hadoop-based PH2 system that enables the exploration of various features of 3D protein structures, e.g., calculation of distances between particular atoms within single protein structures. For this purpose, the system uses the Structured Query Language (SQL)  [21] as a query language, which provides flexibility in querying macromolecular data. 3D protein structures, as raw PDB files, are stored in a replicated way on the Hadoop Distributed File System (HDFS). PH2 system relies on massive parallelism provided by Hadoop computational framework in order to improve the efficiency of the exploration process. SpeeDB reported by Robillard et al. in  [22] also tackles similar problems. It provides in-memory database structure to investigate various types of interactions in proteins, including hydrogen bonds, ionic interactions, disulfide bonds, and aromatic interactions but seems to be less flexible in the exploration capabilities. In-memory protein structure management system was also used by us in the IMPSMS for fast calculations over 3D protein structures  [23]. However, the system suffered the problem of limited memory, leading to a limited number of proteins that could be stored in it.

Since in the paper we show a solution for various types of calculations performed over protein structures that works on the basis of declarative queries, it is also worth mentioning SQL-based approaches for querying protein data stored in relational databases. For example, procedural extensions to Oracle RDBMS for aligning and matching protein sequences, called the ODM BLAST, were reported in [24]. The BioSQL [25], which incorporates modules of the BioJava project [26], focuses on biomolecular sequences and features, their annotation, a reference taxonomy, and ontologies. Several extensions to the SQL language, including PSS-SQL [27,28] and the query language developed by Hammel and Patel [29] and Tata et al. [30], were proposed for searching protein similarities on the basis of protein secondary structures. These works show how protein data can be stored in relational tables. They also present searching techniques that can be applied to explore the data, and how these data are indexed in order to speed up the searching process. Although these extensions allow to operate on the primary (ODM BLAST) and the secondary structures of proteins (the rest), these tools do not directly address processing 3D protein structures. In terms of processing and querying 3D protein structures in relational databases, Mrozek et al. [31] developed the P3D-SQL extension for the Oracle PL/SQL language that allows invoking 3D protein structure similarity searching in SQL queries and performing the process against the whole relational database of 3D protein structures. However, the performance of the solution is worse than the performance of the process executed on raw PDB files stored on hard disc drives (HDD), since the process is executed within Oracle memory pool, which is also limited.

To overcome the problems of limited computational power and limited memory, several cloud-based solutions for exploration processes performed over protein structures were proposed. For example, the system developed by Che-Lun Hung and Yaw-Ling Lin [32] uses Hadoop-based implementations of two popular fold-based alignment methods—DALI [33] and VAST [34]. The  system is scaled on a private cloud. Hadoop and the MapReduce processing model is also used in our previous works [35,36,37] for the same purpose. These works also show that the use of sequential files (instead of processing individual structures) may increase the performance of parallel protein structure similarity searches. Sequential files will also be used in the approach presented in the paper. Dedicated cloud-based architectures were also developed for scalable protein structure similarity searching [38,39,40,41] and protein structure prediction [42,43]. These works prove that cloud resources may significantly simplify and accelerate many calculations related to protein structures.

The approach presented in the paper combines wide scaling capabilities offered by the Cloud computing model, Big Data techniques for efficient data processing, and declarative capabilities of SQL-based solutions that simplify various data explorations by querying Big macromolecular Data  sets.

## 3. Methods and Technologies

To mitigate the above-mention problems we have developed the *PDBUSQLExtractor* that allows parsing and extracting data from PDB files describing structures of proteins, nucleic acids, and their complexes. In this section, we provide implementation details of the scalable parser for the PDB macromolecular data files developed for the Azure Data Lake.

### 3.1. Extensions to the Azure Data Lake Environment

**Figure 1:**
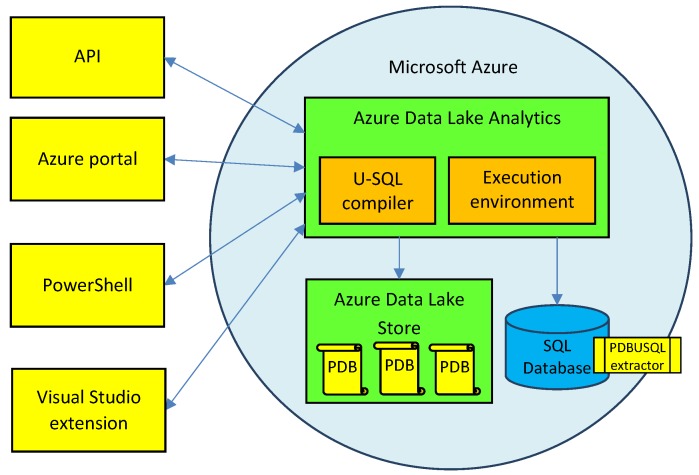
Architecture of our solution for extracting and processing Big macromolecular Data in the Azure Data Lake environment with the *PDBUSQLExtractor*. PDB files describing macromolecular data of proteins and nucleic acids are stored in the Data Lake Store. Efficient processing occurs in the Data Lake Analytics equipped with *PDBUSQLExtractor* library.

Our *PDBUSQLExtractor* parser with all extraction methods was developed for the Azure Data Lake environment. Azure Data Lake (ADL) is a scalable, cloud environment that enables storing and analyzing large data sets. The interactive batch analysis in the ADL is possible in real time for various types of data, including structured, semi-structured, and unstructured data [44]. The architecture of our solution for extracting and processing Big macromolecular Data in Azure Data Lake is presented in Figure 1. The Azure Data Lake consists of two main components:

1. Data Lake Store (DLS), which constitutes a petabyte scale, unlimited storage for a domain-related data lake, in which large collections of data located in various files are distributed across many storage servers. This enables performing read operations in parallel and improves the performance of data read operations.
2. Data Lake Analytics (DLA), which enables efficient and scalable analysis of data stored in Big Data Lakes by parallelizing the analysis on a distributed infrastructure in the Azure cloud. It  provides the U-SQL compiler and distributed execution environment for declarative processing and analysis of data stored in the Data Lake Store.

The U-SQL is a big data query language that combines the declarative capabilities of the SQL query language and expressive power of C# code. Data analysis, preceded by extraction, processing, and transformation of data from the data lake, is performed with the use of the U-SQL scripts containing query expressions. U-SQL scripts can be executed through four available channels, including application programming interfaces (APIs), Azure portal, PowerShell environment, and Visual Studio programming platform. U-SQL programmers and data analysts may use various expressions in data analyses they perform, including the most popular SELECT expression, and also PROCESS, REDUCE, and COMBINE expressions that apply custom or user-defined operators (UDOs). These query expressions produce rowsets that can be assigned to rowset variables. The rowset variables are populated in the EXTRACT U-SQL phrase with the use of appropriate *data extractor*. Built-in extractors, like CSV, TSV, or Text can be used for data extraction. However, specific file formats, like the PDB, require dedicated, custom extractors that know how to extract the data, how a single record looks like, how atomic the information stored in it is, and how to read it. We have developed the *PDBUSQLExtractor* for parsing and extracting data from PDB macromolecular data files describing structures of proteins and nucleic acids. Custom extractors, as .NET assemblies are stored in the Azure SQL Database. Each execution of the U-SQL script invoking the *PDBUSQLExtractor* causes loading the extractor-related DLL library into the execution environment of the Azure Data Lake Analytics. Macromolecular data of proteins and nucleic acids are stored in the Azure Data Lake Store. The Data Lake Analytics has wide access to the data stored in the repository (DLS). Results of data extraction and processing are also stored in the Data Lake Store by invoking the U-SQL OUTPUT expression that uses appropriate *data outputter*. Likewise in the EXTRACT phase, data outputters can be built-in or  custom.

### 3.2. Setting Up the Azure Data Lake for Scalable Extraction of Macromolecular Data

To use the *PDBUSQLExtractor* a user must initially complete 3 steps:

1. set up the Azure Data Lake environment,
2. upload PDB files with macromolecular structures into the Azure Data Lake Store,
3. register the *PDBUSQLExtractor* library in the Azure Data Lake Analytics.

All of the mentioned operations can be completed through the Azure portal, Azure PowerShell, Azure CLI, or appropriate Application Programming Interfaces (APIs). Users should follow the Azure Data Lake Analytics Documentation [45] for the particular method. Setting up the Azure Data Lake environment in the Microsoft Azure cloud (step 1) can be easily done through the Azure portal (http://portal.azure.com/), which provides a graphical interface that simplifies the management and configuration of the ADL platform. The portal is available through web browsers in any operating system (OS). What is important, users must set up the ADL environment within their own Azure subscriptions, since they will bear the costs of using the platform (costs of storing the data and costs of computations performed). The same portal can be used for uploading the processed and analyzed data describing macromolecular structures (step 2). However, in the case of many files with macromolecular data, the preferable way may be using one of the command-line tools, like the Azure PowerShell or the Azure CLI. Azure PowerShell is an extension of Windows PowerShell that provides cmdlets for simplifying and automating the management of Azure cloud services. Azure CLI is another Microsoft’s command-line tool for managing Azure resources, but in contrast to the Azure PowerShell, it can operate on various OS platforms. Registering the *PDBUSQLExtractor* library (*PDBUSQLExtractor.dll*, please refer to the Availability section at the end of the paper) in Azure Data Lake environment (step 3) is necessary for using the created data extractors in U-SQL scripts, since the library extends standard capabilities of the ADL environment. The library must be registered as an assembly in one of the Azure SQL Databases (*master* by default) available in the Azure Data Lake environment. Code editors, like Microsoft Visual Studio, greatly facilitate the library registering task, though all mentioned methods can be used for this.

After completing the three necessary steps, the user can start developing the U-SQL scripts for data extraction and secondary data analysis. For the development of the U-SQL scripts, he can use any code editor, preferably Microsoft Visual Studio or Visual Studio Code. The latter one is an open-source and free source code editor developed by Microsoft for Windows, Linux, and macOS. Both editors include support for debugging, embedded Git control, syntax highlighting, intelligent code completion, snippets, and code refactoring. The developed U-SQL scripts can be executed in the Cloud, in the configured Azure Data Lake environment, or tested locally. Cloud executions allow for parallelization of computations related to data extraction and secondary analysis and scaling the computations on many compute units. Local executions, used for testing the U-SQL scripts, are supported by Azure Data Lake Tools for Visual Studio or by Azure Data Lake U-SQL SDK.

### 3.3. Modules and Methods for Parsing PDB Files

In the U-SQL scripts, parsing and extracting of macromolecular data of proteins and nucleic acids is possible by using a dedicated extractor from the created class PDBUSQLExtractor. The extractor is invoked in the EXTRACT U-SQL statement as it is shown in Listing 1 in line 22. In the presented data extraction, we parse and extract data from the ATOM sections of PDB files (*.ent) located in the indicated folder in the Data Lake Store. The dedicated extractor parses each *.ent file in the folder and fetches data from an appropriate section. At the moment, we have implemented the extraction process for the following sections of the PDB files:

- ATOM that presents the atomic coordinates for standard amino acids and nucleotides. It also presents the occupancy and temperature factor for each atom.
- HETATM that presents non-polymer or other non-standard chemical coordinates, such as water molecules or atoms presented in HET groups, together with the occupancy and temperature factor for each atom.
- SHEET that is used to identify the position of β-sheets in protein molecules.
- HELIX that is used to identify the position of α-helices in protein molecules.
- SEQRES that contains a listing of the consecutive chemical components (amino acids for proteins) covalently linked in a linear fashion to form a polymer.

Although we developed the *PDBUSQLExtractor* extractor mainly for parsing PDB files with macromolecular data describing 3D structures of proteins, it can be also used to extract data from PDB files describing nucleic acids and DNA/RNA-protein complexes. For the DNA/RNA molecules, it is possible to extract data from the ATOM, the HETATM, and the SEQRES sections.

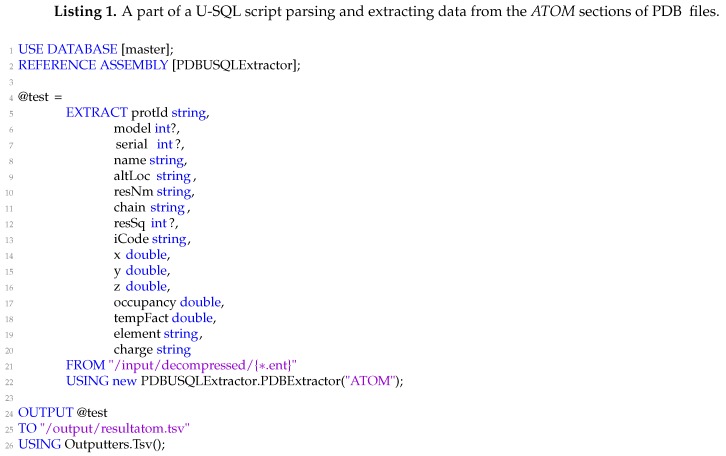



**Figure 2:**
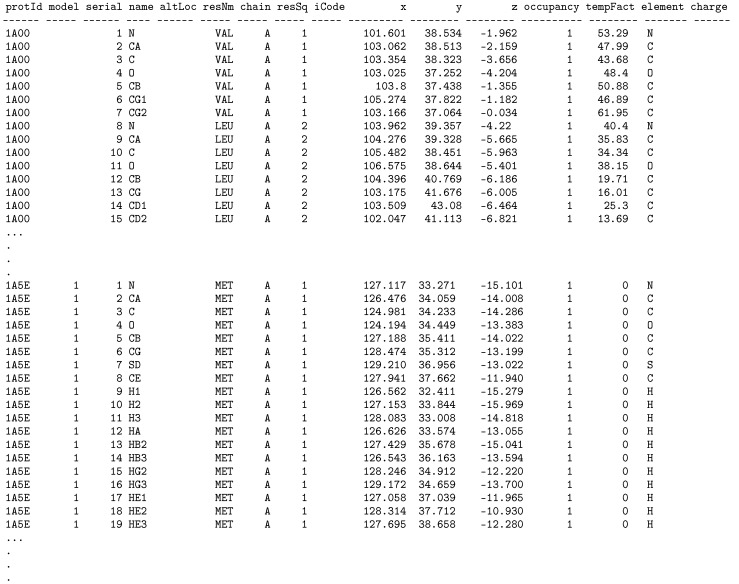
Results of the extraction of the ATOM section performed with the U-SQL script from Listing 1 showing a part of produced rowset.

Attributes fetched during the execution of the extraction script and data types for the attributes are specified dynamically in the EXTRACT clause of the U-SQL statement (lines 5–20). They should be adjusted to the section of the PDB files that is currently extracted. While developing our dedicated extractors we followed the PDB file format specification [46]. Execution of the whole EXTRACT statement produces the @test rowset (line 4) that can be further processed and analyzed (example will be shown in Section 4) or stored in the Data Lake Store for future processing. A part of the rowset produced by the presented U-SQL script has the form shown in Figure 2 (column headers were formatted manually). Storing the produced rowset is performed in the OUTPUT statement (lines 24–26).

Similar scripts can be implemented to extract other sections of the PDB macromolecular data files for proteins, nucleic acids, and nucleic acid-protein complexes. For example, Listing 2 shows a sample script for parsing and extracting data from the SEQRES sections of PDB files. The only difference from the previous script is the SEQRES value of the parameter of the *PDBExtractor* used in the USING clause of the EXTRACT statement (line 23) and attributes fetched in the EXTRACT clause (lines 5–21). The data from the SEQRES records of the PDB files are extracted according to the PDB file format documentation and the list of attributes contains: a serial number of the SEQRES record for the current chain (serNum), chain identifier (chainId), number of residues in the chain (numRes), and names of residues returned in thirteen following columns (resName1–resName13). Each returned row in the produced rowset (@test, line 4) is preceded by the protein PDB ID identifier (proteinId) since we process many PDB files.

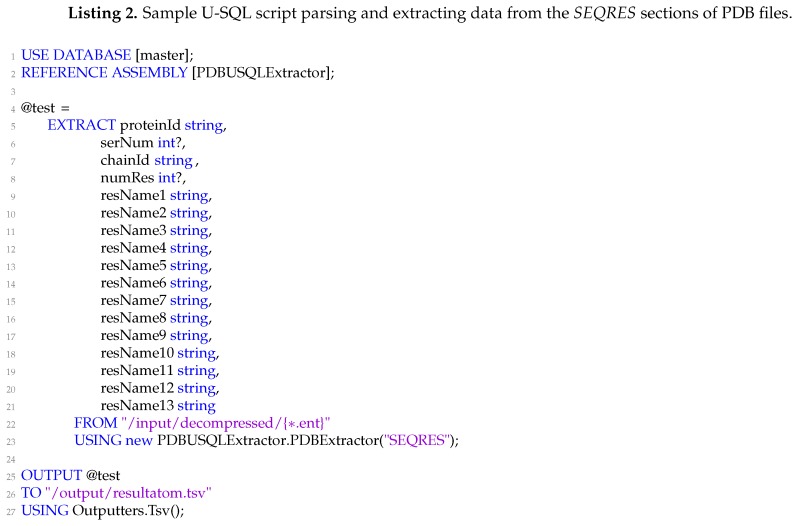



**Figure 3:**
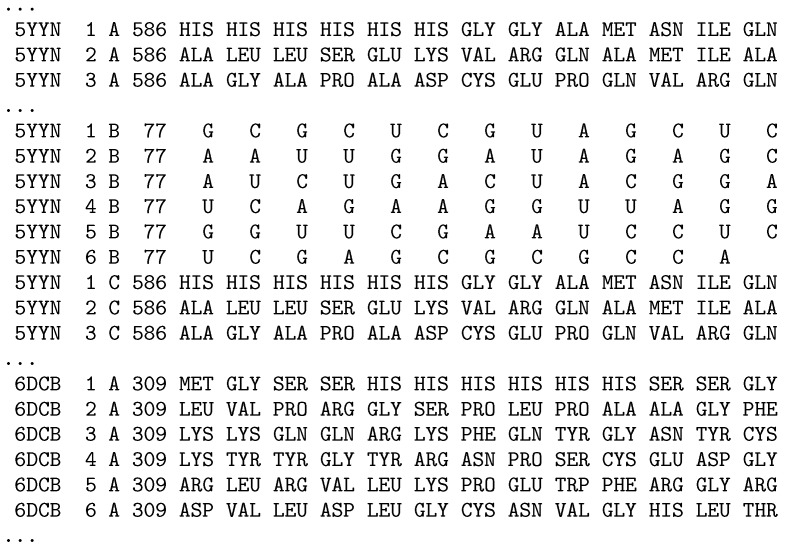
Results of the extraction of the *SEQRES* section performed with the U-SQL script from Listing 2 showing a part of produced rowset.

Execution of the sample script produces the @test rowset (line 4). Attributes of the rowset that are needed for further analysis can be selected in the SELECT U-SQL expression (example will be shown in Section 4). A part of the rowset produced by the presented U-SQL script has the the form shown in Figure 3.

### 3.4. Optimizations

The *PDBUSQLExtractor* parser and extractor was developed in such a way that it supports space-optimized and performance-optimized storage of macromolecular data in the Cloud. In terms of use of storage space for macromolecular data, the extractor allows to work with:

- decompressed PDB macromolecular files,
- compressed PDB macromolecular files.

Using the compression allows reducing the storage space consumed on the Cloud, which has a positive impact on the costs of the Cloud storage. The *PDBUSQLExtractor* supports the standard *.gz compression for the PDB files. However, those users that possess the collections of decompressed macromolecular data in the form of PDB text files may also use the parser in data extraction and  processing.

In terms of performance of the parsing and data extraction process, the *PDBUSQLExtractor* works on two types of data:

- individual macromolecular data files that are extracted in parallel,
- joined, sequential macromolecular data files that are also extracted in parallel.

We assume that in a regular approach the user operates on the repository of standard, individual PDB macromolecular data files. These files will be extracted in parallel according to the parallelism factor set by the user while executing the U-SQL script in the Azure Data Lake environment. However, we also noticed that much better performance can be gained while processing the macromolecular data assembled in sequential macromolecular data files of larger sizes. Therefore, we also gave the possibility to process the data in such a way for these users that prioritize the performance.

**Figure 4:**
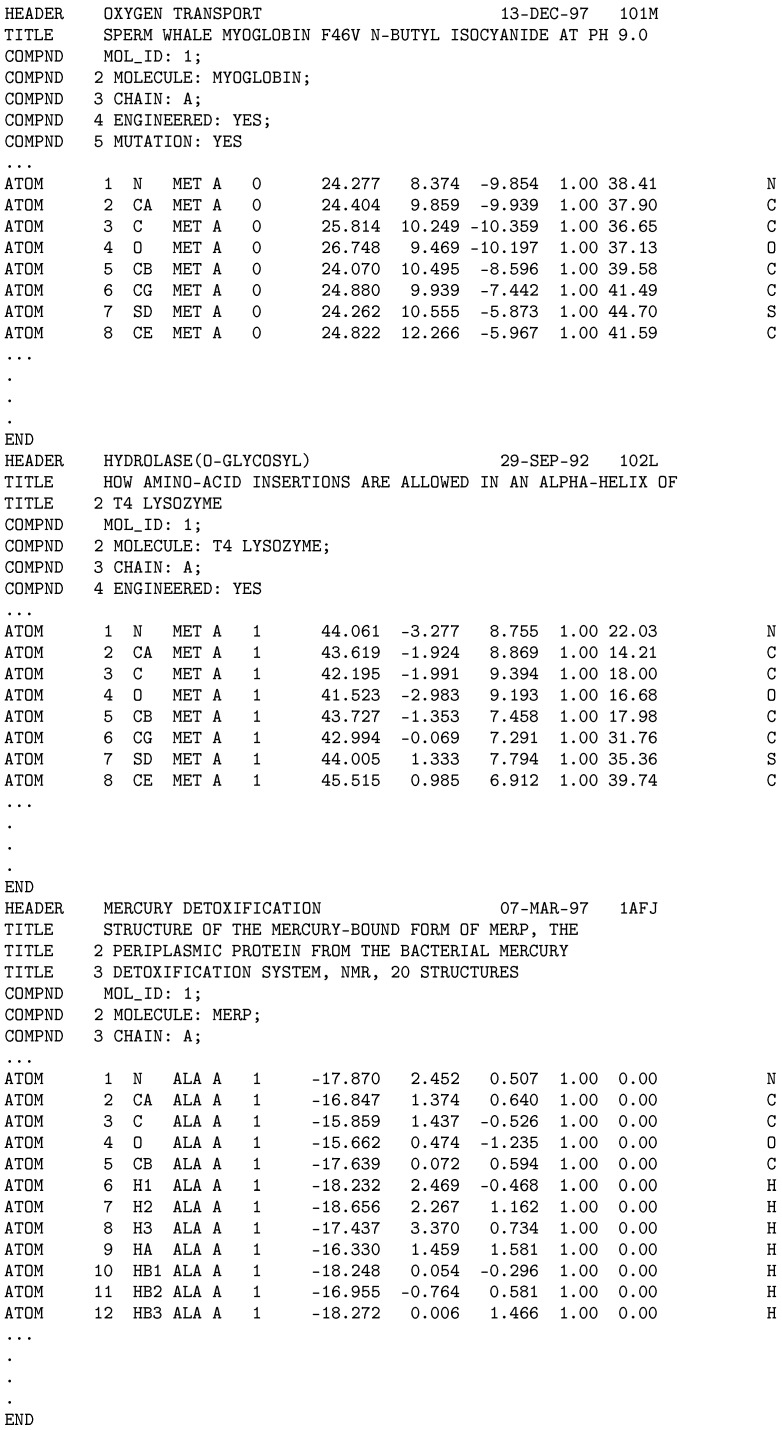
A part of a sample sequential file with many protein structures stored as records for extraction by the *PDBUSQLExtractor* parallel parser. Each END section determines the border for a single record processed in the U-SQL script for Data Lake Analytics.

A part of a sample sequential file with several protein structures (in the PDB format) treated as single records in the ADL-based extraction and parsing is shown in Figure 4. The developed extraction method of the *PDBUSQLExtractor* parser uses the END section of each PDB data set to recognize the end of the record in the sequential file. Such a sequential file can be simply produced by using the *copy* command of the Microsoft Windows command line console.

Extraction of the sequential files is performed with the use of the dedicated PDBConcatExtractor extractor, invoked in the USING clause of the EXTRACT statement in the U-SQL processing script, as it is presented in Listing 3, line 8. The extractor accepts the same parameters as the PDBExtractor extractor used for processing macromolecular data stored in individual PDB files.

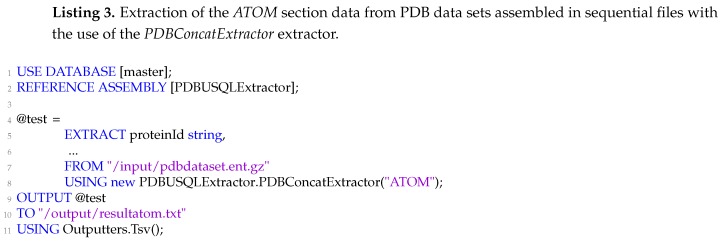



The advantage of the processing with the use of sequential files is that compute units used by the Data Lake Analytics for parallel extraction and processing will be initiated once. The initiation time is negligible in relation to the extraction and processing time of macromolecular data. The limitation of this solution is the maximum size of the row (record) the extractor can process, which is fixed to 4 MB. This means that the input collection should be divided into files of the size larger than 4 MB and those that are smaller than 4 MB. The smaller files should be concatenated into larger sequential files, and the larger ones should be extracted individually.

Both types of files, individual and sequential, can be also stored as compressed and decompressed in the Azure Data Lake Storage and processed in the Data Lake Analytics. Influence of this optimization on the performance of the extraction will be studied in Section 4.

## 4. Results

Azure Data Lake allows parallelizing many jobs related to data processing. Parallelization of macromolecular data extraction should bring significant improvements in the performance of the *PDBUSQLExtractor* and reduction of the execution time. We tested the reduction of the execution time in a series of experiments performed in the Azure Data Lake environment.

### 4.1. Experimental Setup

The performance of the *PDBUSQLExtractor* was tested with the use of macromolecular data of 3D protein structures taken from the Protein Data Bank. We used two data sets in our tests:

- data set DS1 containing 1475 files,
- data set DS2 containing 14,750 files.

The amount of data constituted  1% (DS1) and  10% (DS2) of 3D structures stored in the repository at the time of experiments. Data extractions were performed for three different sections of PDB files,  i.e.,:

- ATOM,
- SHEET,
- SEQRES.

Tests were performed with compressed and decompressed PDB macromolecular files stored as individual files and sequential files in all combinations. This gave four scenarios of performed  experiments:

- decompressed-individual files (DI),
- compressed-individual files (CI),
- decompressed-sequential files (DS),
- compressed-sequential files (CS).

Parallelization was regulated by the *parallelization factor*, which was changed from one to 1475. This means that in the performance experiments we changed the number of compute units, in Azure Data Lake called *Allocation Units* (AUs), performing extractions and calculations from one up to 1475 (exactly, #AU=1,2,8,32,128,512,1024,1475).

To compare obtained results with traditional, local processing, we also used a PC workstation with CPU Core i7 4700MQ 2.4GHz (4 cores, 8 threads), RAM 16GB, storage HDD 1TB, working under control of the Microsoft Windows 7 64-bit operating system for performing pure extractions of ATOM sections of processed data sets.

### 4.2. Execution Times

In the first series of tests, we verified the performance of the extraction process for 3D protein structures stored separately in individual files. This is the standard format that scientists from the whole world can use when downloading macromolecular data of proteins from the Protein Data Bank repository. In this series of tests, we used the data set DS1 with 1475 protein structures (files) and scaled the extraction process from 1 up to 1475 allocation units (AUs) used by the Azure Data Lake while executing U-SQL extraction scripts. Results of these tests are presented in Table 1.

**Figure 5:**
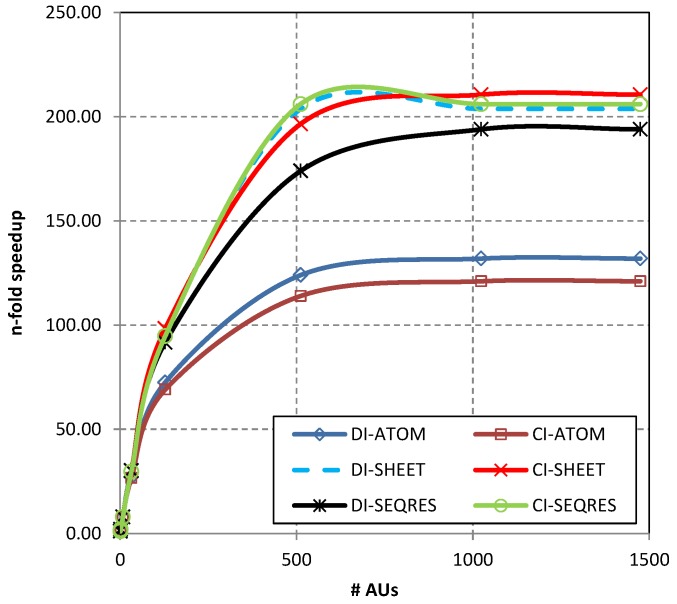
n-fold speedups achieved when extracting data from various sections (ATOM, SHEET, SEQRES) of compressed (CI) and decompressed (DI) individually stored PDB files for the varying number of allocation units (AUs).

As can be observed in Table 1 the execution time gradually decreases with the number of AUs (increasing parallelization factor). The execution time consists of the extraction time, the time needed for aggregating data from parallel streams, and the time of storing results. For a single AU, it takes more than 3 hours to extract appropriate sections and store extracted data in a new CSV file. The  execution time is much better for 32 AUs—the extraction, aggregation, and storing data takes between 6 and 8  min (391 s to 459 s, depending on the section and compression used). For 1024 AUs the process takes less than 2 min (e.g., 97 s for the CI-ATOM). Extraction of the ATOM sections takes more time than the extraction from other sections. This is due to the larger amount of data that must be extracted, aggregated, and finally, stored after the extraction. Generally, the extraction phase is parallelized better than aggregating and storing data. Therefore, speedup gains are not huge for the increasing number of used AUs. Shapes of the n-fold speedup curves can be observed in Figure 5.

**Figure 6:**
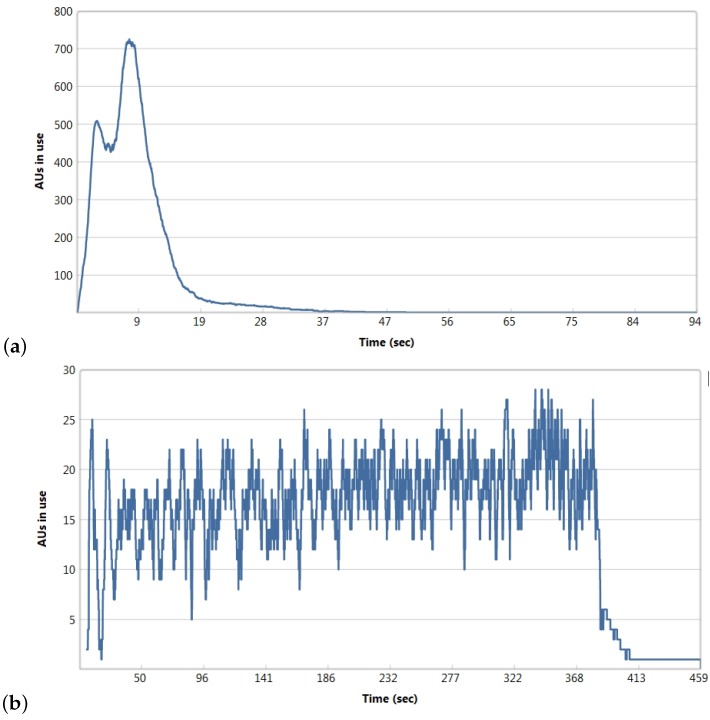
Use of compute resources (AUs) during the whole extraction process on data set DS1 for (**a**) 1024 AUs, and (**b**) 32 AUs assigned to the execution.

More details on the use of assigned compute resources can be seen in Figure 6. Figure 6a shows that after parallelizing the process on 1024 AUs most of the AUs are used only in the extraction phase in the first 20 s. The aggregation of extracted data and storing takes the rest of the 94 s taken by the whole process. When assigning less AUs, the use of the resources is more effective. For example, for the 32 AUs assigned, most of the resources are used through the whole execution process (until the 400th s, Figure 6b).

Compression of data has almost no effect on the execution time. Nevertheless, the compressed data are extracted a little bit faster than uncompressed data in most cases (e.g., 459 s for DI-ATOM and 441 s for CI-ATOM, for 32 AUs in use, see Table 1). The compression, however, has a significant impact on the storage space occupied. For example, the compressed data set DS1 used in this series of tests occupied 139 MB, and the decompressed one occupied 614 MB.

Unfortunately, we could observe that the operating time was generally long for a relatively small total data size. Although the extraction of individual PDB files is well-parallelizable, it appeared to be a time-consuming process, especially for the executions that use a small number of AUs. The  same extraction process performed on the PC workstation took between 7 and 9 min (Table 1, the PC column). This execution time is comparable to the time achieved by 32 AUs processing the same data set DS1 on the Azure Data Lake. This shows that processing individual files in highly scalable Big Data environment is possible, but not impressive in terms of execution times. There are two factors deciding about this. First of all, PDB files must be analyzed atomically. This means that the Azure Data Lake cannot split the analysis of one file between several AUs, because the relevant information, such as model number and protein ID, are stored in other sections of the file. The second factor is the specificity of the Data Lake Analytics platform. It is adapted and optimized to process large data sets. The internal implementation of the ADLA development engine favors the code written directly in the U-SQL from the code written through extensions in the C# language. This is due to the fact that the U-SQL code is compiled to the C++ language and the managed code is executed in the Common Language Runtime (CLR) virtual machine. The CLR virtual machine initialization process is relatively long. The larger the data size, the more negligible the process is. Unfortunately, in the series of tests, the average file size taken from the Protein Data Bank was 96 KB for compressed files and 417 KB for uncompressed files. Initialization of CLR virtual machines consumes most of the time of AU units. At  the same time, the ADL management process allocates one analytical unit for one input stream. This results in a huge time surplus dedicated to the initialization, finalization, and cleaning of CLR machine artifacts.

The proposed solution for this problem is to pre-process the macromolecular data and combine multiple files into sequential files, as we proposed in Section 3.4. In this case, each component PDB file of the sequential file can be treated as a separate input row for the extractor. Another advantage in this approach is the ability to opt out of the atomic processing of the sequential file. Then, compute units of the Azure Data Lake Analytics have to be initialized only once, which should additionally reduce the execution time. We checked this approach in the second series of tests.

For the second series of experiments, we created one sequential file with all macromolecular data from the data set DS1. Then, we run the data extraction process again in the Azure Data Lake. It was enough to use only one AU for the extraction process. Results of the execution are presented in Table 2. The execution time of the extraction, aggregation, and storing data in output files takes less than 2 min (116 s) when extracting the ATOM section of the PDF files, and less than 1  min when extracting data from the SHEET (46 s) and the SEQRES (51 s) sections. When processing individual PDB files, such a short execution time is achievable when using hundreds of AUs (see execution times in Table 1 for 128 and 512 AUs). This confirms that the Azure Data Lake and our *PDBUSQLExtractor* are adjusted to processing Big Data files.

Again, the compression (CS) has no effect on the execution time, but on the storage space occupied by the sequential files. The occupied storage space was similar to the one provided for individual files, as the files were just concatenated.

In the third series of experiments, we decided to use a larger set of protein structures. For this purpose, we used the data set DS2 with 14,750 protein structures, randomly selected from the Protein Data Bank. The set of proteins was arranged in 10 sequential files of similar sizes. The parallelization factor was changed from 1 to ten (we used up to 10 AUs) during the data extraction. Results are presented only for the extraction of the atomic positions from the ATOM section, as the extraction of this section was the longest one among all the sections that were processed.

**Figure 7:**
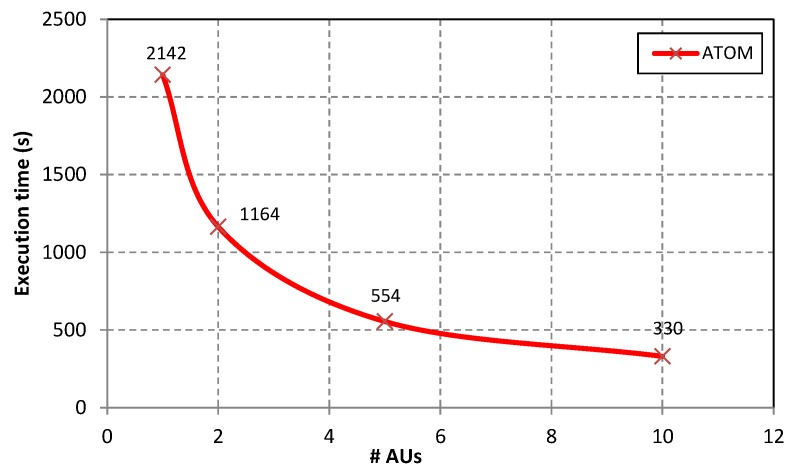
Execution time when extracting data from the *ATOM* section of PDB files stored in 10 sequential files (data set DS2) for the varying number of allocation units (AUs).

Results of the execution of the whole extraction from the ATOM section of PDB files stored in sequential files for the varying number of allocation units (AUs) is presented in Figure 7. As can be observed the best execution time was achieved when extracting atomic data from 10 sequential files storing approximately 10% of the Protein Data Bank content with the use of 10 AUs—the process took only 330 s.

This confirms that our optimization in storage format by combining multiple PDB files into sequential files, and development of appropriate U-SQL data extractor, gives measurable benefits for the efficiency of the extraction process. The execution time has significantly decreased when comparing it to processing individual files (see Table 1), and the cost of the processing according to the Data Lake Analytics price list has been significantly reduced (in the Cloud users pay for the time of used compute resources). The execution time of the U-SQL scripts became also more predictable compared to the first solution.

### 4.3. Sample Calculations

In this section, we show sample calculations that are accompanied by the extraction of atomic positions. In the presented example, we calculate distances between successive $C_{\alpha }$ atoms in each chain of successive proteins. Then, we select only these residues for which the distance to $C_{\alpha }$ atoms in the following residue is around 3.81Å. For two $C_{\alpha }$ atoms from a protein chain:

(1) $$a_{i}^{C_{\alpha }}={\left(x_{i},y_{i},z_{i}\right)}^{T}\;\mathrm{and}\;a_{i+1}^{C_{\alpha }}={\left(x_{i+1},y_{i+1},z_{i+1}\right)}^{T},$$

where x,y,z are Cartesian coordinates of particular $C_{\alpha }$ atoms, the distance between them can be calculated as the norm:

(2) $$d_{i,i+1}^{C_{\alpha }}=∥a_{i}^{C_{\alpha }}-a_{i+1}^{C_{\alpha }}∥=\sqrt{{\left(a_{i}^{C_{\alpha }}-a_{i+1}^{C_{\alpha }}\right)}^{T}\,\left(a_{i}^{C_{\alpha }}-a_{i+1}^{C_{\alpha }}\right)},$$

or by using the Euclidean distance:

(3) $$d_{i,i+1}^{C_{\alpha }}=\sqrt{{\left(x_{i+1}-x_{i}\right)}^{2}+{\left(y_{i+1}-y_{i}\right)}^{2}+{\left(z_{i+1}-z_{i}\right)}^{2}}.$$

Unless the data on the positions of $C_{\alpha }$ is prepared, the extraction process precedes the calculations and the *PDBUSQLExtractor* must be used. Listing 4 shows a sample U-SQL script that may implement the whole data processing task.

**Figure 8:**
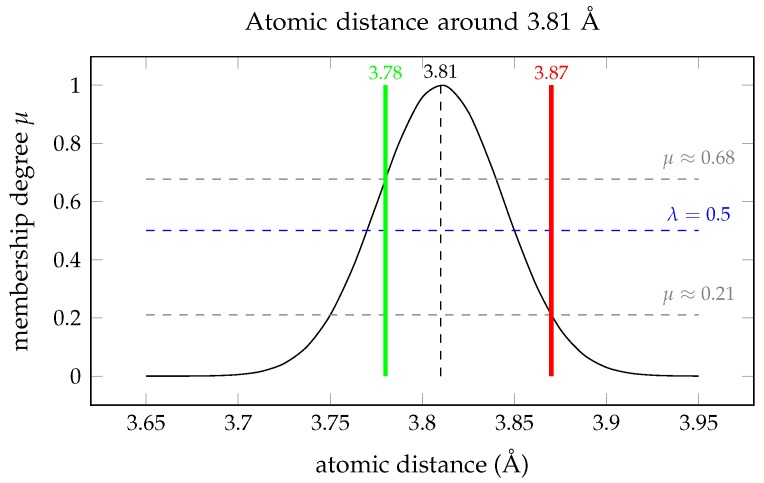
Visual interpretation of fuzzy selection with the fuzzy search condition *Atomic distance around 3.81 Å* showing two values of the inter-atomic distance in protein structures: 3.78 that satisfies the condition (μ(3.78)>λ), and 3.87 that does not satisfy the condition (μ(3.87)<λ) for λ=0.5.

The U-SQL script references two external libraries. The *PDBUSQLExtractor* library contains implementations of extractors for individual and sequential PDB files (line 1). The FuzzySearchLib library (referenced in line 2) contains modules with classes and methods that allow using fuzzy search conditions to find all those distances that are around 3.81Å. Data extraction is performed by the EXTRACT expression executed in section S1 of the script (lines 6–25). The expression uses the PDBConcatExtractor (line 25) for fetching data from the ATOM sections of the PDB files assembled in a sequential file indicated in the FROM clause (line 24). Appropriate data from the ATOM sections of each protein, including the x,y,z coordinates of atoms, are extracted and supplemented by the proteinId and modelId (lines 8–9). The SELECT expression in section S2 of the U-SQL script (lines 27–39) retrieves only these fields of the produced rowset that are further needed for the calculations (lines 29–37), and only these rows of the rowset that correspond to the $C_{\alpha }$ atoms by applying appropriate filtering condition in the WHERE clause (line 39). The SELECT expression in section S3 of the U-SQL script performs the calculation of distances between two successive $C_{\alpha }$ atoms in each protein chain and produces the new rowset with distances, called @filteredDistances. Finally, the rowset is used in the SELECT expression in section S4 of the U-SQL script (lines 56–61). This expression implements a fuzzy query with the fuzzy search condition *Distance around 3.81Å*. The *Udfs.Around* function processes *CaCaDistance* values and compares them flexibly with the fuzzy set *Atomic distance around 3.81Å* defined by the Gaussian membership function presented in Figure 8. The fuzzy selection in the WHERE clause accepts only those residues that satisfy the fuzzy condition *CaCaDistance around 3.81* with a minimum degree of truth (similarity or membership degree) λ=0.5 (line 61). Visual interpretation of the fuzzy selection is presented in Figure 8.

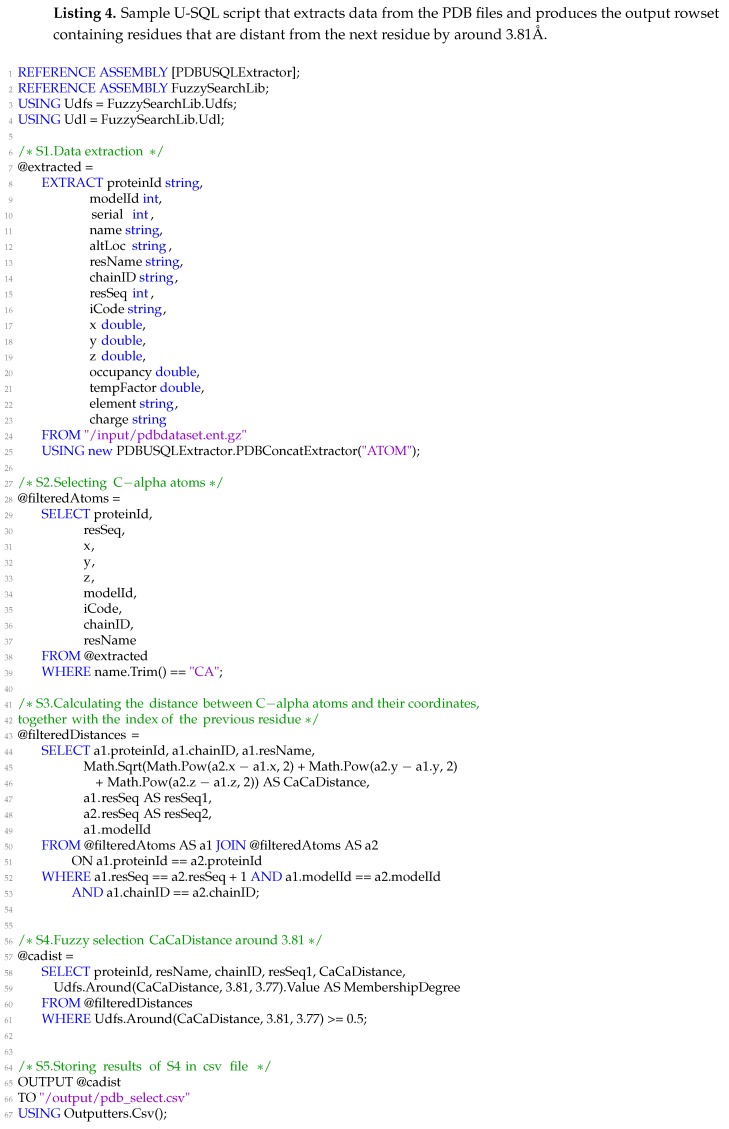



**Figure 9:**
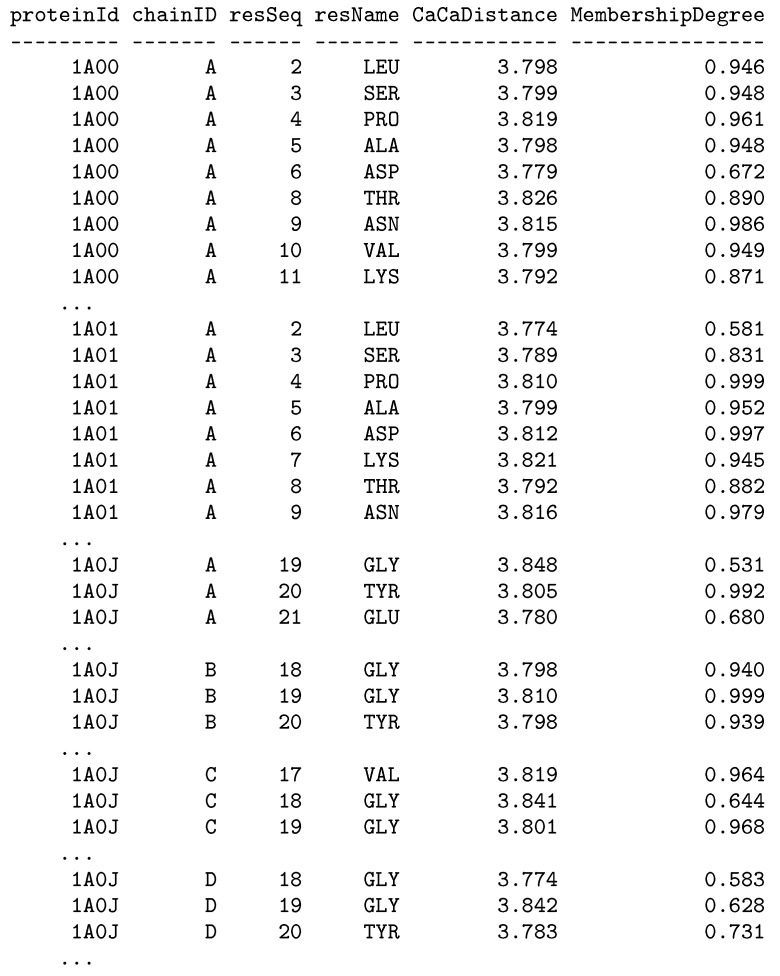
Results of the extraction and the analysis performed with the U-SQL script from Listing 4 showing a part of produced rowset.

Such a U-SQL code generates the report shown in Figure 9, which is saved to CSV files in the Azure cloud storage by executing the OUTPUT statements S5 in lines 65–67. This report shows residues with values of the Cα–Cα distance around 3.81Å accompanied by membership degrees, which constitute degrees of truth how the particular distance value matches the fuzzy selection condition.

**Figure 10:**
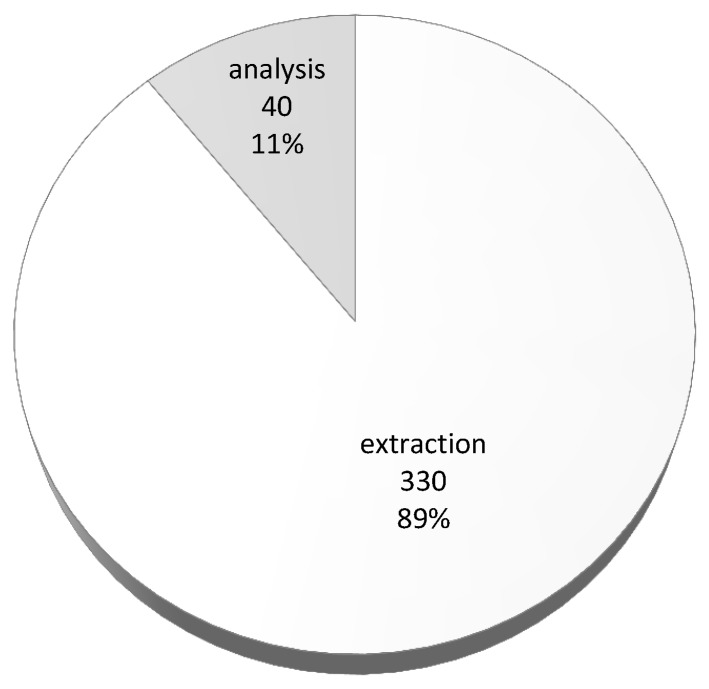
Computational costs of data extraction and data analysis for the sample U-SQL script executed for proteins from the data set DS2 stored in ten sequential files with 10 AUs.

The execution of the presented U-SQL script for 14,750 protein structure from the DS2 data set stored in ten sequential files with 10 AUs took 370 s. In Figure 10 we can see the computational costs of the data extraction and the data analysis. We can see clearly that, in case of the presented analysis encoded in the U-SQL script, the computational cost of the data extraction is much higher (330 s) than the computational cost of the analysis performed (40 s). However, the proportions depend on the type of the analysis and complexity of the U-SQL code nested between the EXTRACT and the OUTPUT expressions.

## 5. Discussion

Efficient parsing and extracting of information from macromolecular data files describing 3D structures of proteins, nucleic acids, and nucleic acid-protein complexes is important for performing various sophisticated calculations on the structures on large scale. This will be especially attractive for all specialists working in the field of structural bioinformatics, computational biology, and drug design. The solution presented in the paper satisfies the requirements of the mentioned groups of specialists two-fold: (1) by providing a huge storage space for macromolecular data in the Data Lake Store in the Azure cloud, (2) by enabling scalable, parallel calculations on macromolecular structures in the Data Lake Analytics.

Results of performed experiments confirmed that the presented data extractors can be successfully used in retrieving data from particular sections of the PDB files stored in a variety of ways—(1) as individual compressed or decompressed files, or (2) as sequential compressed or decompressed files. The first approach does not require any initial processing of the data—they can be loaded to the cloud storage space as they are retrieved from the Protein Data Bank repository servers or from the local equipment or installed software tools, compressed or decompressed. Compression does not cause significant delays in data extraction in the Data Lake Analytics, but allows to save the storage space. The second approach requires some preprocessing prior to storing the data in the cloud storage space—sequential files must be created by linking many smaller PDB files into larger ones. This preprocessing may take some small amount of time, but causes that the extraction process is extremely fast comparing it to the one performed with individual files. This allows to extract more macromolecular data with a lower parallelization factor (less compute units are used) and, as a consequence, to reduce the costs of data processing in the Cloud platform. The results of our performance tests show that an important factor when working with the Azure Data Lake platform is to design tasks according to the specificity of the platform and its characteristics. The platform is intended to operate on large input, which favors processing sequential macromolecular data files, rather than individual ones. In less than two minutes, the platform with one compute unit (allocation unit) is able to extract and process the sequentially stored macromolecular data of proteins, which would be extracted by hundreds of compute units for the same number of proteins stored individually.

Our solution complements various calculators for atomic interactions or inter-residue contacts presented in Section 2. It is less specific in terms of performed operations, but is more flexible. It does not provide any ready solution to calculate the particular type of interaction. However, users are able to encode their calculations in the U-SQL scripts after performing the extraction process. The U-SQL language gives the flexibility that allows performing any operation on macromolecular data in parallel once the data is extracted. This is completely different than the tools that provide dedicated, friendly, but closed web front-ends. In terms of the user interface, the presented solution is similar to the one presented in the PH2 system, but it is based on a different computational framework. The PH2 system uses Hadoop, while our solution is based on the Azure Data Lake. It is less efficient than in-memory solutions, like SpeeDB and IMPSMS, but gives more freedom in managing the underlying data and have a friendly U-SQL interface giving flexibility in various calculations.

Among important advantages of the presented solution, it is also worth noting that it can be broadly scaled out in the Azure cloud for petabytes of macromolecular data that we are awaiting due to the exponential growth of data in the field of bioinformatics. As a disadvantage, we should mention that the *PDBUSQLExtractor* is able to operate only within the Azure Data Lake platform. It is not possible to use it in other data processing platforms, like Apache Hadoop or Apache Spark. This flows mainly from the programming model of the Azure Data Lake, which *PDBUSQLExtractor* was adjusted to. Another disadvantage is the necessity to pay for the used storage space and, especially, for used compute resources of the Cloud platform. However, according to the principles of Cloud computing, users have to pay only for what they use and are excepted from the maintenance of the hardware. Taking into account current rates and unavoidable dynamics of the data growth in bioinformatics, we have to prepare efficient tools for such situations and our solution perfectly fits this scenario.

Future works will be focused on further development of the presented data extractors that would enable parsing and extracting data from other file formats, like mmCIF and PDBML. We also encourage readers to use the *PDBUSQLExtractor* while performing various sophisticated calculations on macromolecular structures in their research works. The Azure Data Lake also seems to be a good environment for performing efficient analyzes of other types of biological data, including data obtained in Next Generation Sequencing or mass spectrometry-based proteomics experiments. We are carrying works on the use of the platform in these areas of bioinformatics as they need such scalable solutions due to the enormity of data.

## 6. Availability

The *PDBUSQLExtractor* library is available at GitHub (https://github.com/dmrozek/repo-PDBUSQLExtractor) and at the project homepage http://zti.polsl.pl/w3/dmrozek/science/pdbusqlext.htm. The library can be used by the academic society for free, i.e., the software is free, but users have to set up their own Azure Data Lake execution environment in the Azure cloud to use the library for processing and analysis of their own data. Setting up steps are provided at the *PDBUSQLExtractor* project home page.

Further development of the system will be carried out by the Cloud4Proteins non-profit, scientific group (http://www.zti.aei.polsl.pl/w3/dmrozek/science/cloud4proteins.htm).

## Figures and Tables

**Table 1:** Execution time (s) for local (PC) and distributed (Azure Data Lake) extractions of various sections of the PDB files and the varying number of allocation units (AUs) for 3D protein structures stored separately as compressed (CI) and decompressed (DI) individual files.

Azure Data Lake									PC
**# AUs**	**1**	**2**	**8**	**32**	**128**	**512**	**1024**	**1475**	**1**
DI-ATOM	12,400	6225	1606	459	171	100	94	94	464
CI-ATOM	11,740	5892	1526	441	170	103	97	97	516
DI-SHEET	12,225	6115	1548	412	128	60	60	60	–
CI-SHEET	11,793	5898	1484	391	120	60	56	56	–
DI-SEQRES	11,831	5922	1494	393	129	68	61	61	–
CI-SEQRES	11,746	5879	1482	396	124	57	57	57	–

**Table 2:** Extraction time (s) for various sections of the PDB files for 3D protein structures stored in compressed (CS) and decompressed (DS) sequential files (data set DS1) for the execution with one allocation unit (AU).

	ATOM	SHEET	SEQRES
DS	116	46	51
CS	116	46	51

## Abbreviations

The following abbreviations are used in this manuscript:

ADL	Azure Data Lake
API	Application Programming Interface
AUs	allocation units
CI	compressed-individual files
CLR	common language runtime
CS	compressed-sequential files
CSV	comma-separated values
DI	decompressed-individual files
DLA	Data Lake Analytics
DLL	Dynamic-Link Library
DLS	Data Lake Store
DS	decompressed-sequential files
DS1	data set 1
DS2	data set 2
IMPSMS	In-Memory Protein Structure Management System
OS	operating system
PDB	Protein Data Bank
SDK	Software Development Kit
SQL	Structured Query Language
TSV	tab-separated values
UDO	user-defined operator
VMs	virtual machines
